# SAPHO Syndrome Complicated by Lesions of the Central Nervous System Successfully Treated with Brodalumab

**DOI:** 10.1155/2023/6005531

**Published:** 2023-02-09

**Authors:** Masahide Funabiki, Masayuki Tahara, Seiko Kondo, Naho Ayuzawa, Hidetoshi Yanagida

**Affiliations:** ^1^Department of Rheumatology, National Health Organization Utano National Hospital, Kyoto, Japan; ^2^Department of Clinical Immunology and Rheumatology, Medical Research Institute KITANO HOSPITAL, PIIF Tazuke-Kofukai, Osaka, Japan; ^3^Department of Neurology, National Health Organization Utano National Hospital, Kyoto, Japan

## Abstract

Synovitis-acne-pustulosis-hyperostosis-osteitis (SAPHO) syndrome is a rare disease with an unknown entity that affects the skin and the peripheral and/or axial joints. Here, we report on a patient with SAPHO syndrome complicated by lesions of the central nervous system who was successfully treated with brodalumab, an IL-17 receptor blocker. He had been suffering from arthralgia in the wrists and knees as well as axial symptoms such as back pain and assimilation of cervical vertebrae. He had been treated with corticosteroid, salazosulfapyridine, methotrexate, and bisphosphonate; however, his peripheral and axial articular manifestation were intractable. Recently, biologics predominantly targeting TNF-*α* is employed for difficult-to-treat SAPHO cases; however, he had been complicated with the lesions of the central nervous system resembling multiple sclerosis (MS), an inflammatory demyelinating disorder in the central nervous system, for which application of TNF-*α* inhibitor is contraindicated. Alternatively, brodalumab was administered , which promptly ameliorated the articular manifestations without aggravating the lesions of the central nervous system. We propose that this type of IL-17 blockade could be an alternative therapy for DMARDs-resistant SAPHO syndrome.

## 1. Introduction

SAPHO syndrome is a chronic inflammatory disorder that compromises the skin, joints and bones. The disease is characterized by characteristic skin symptoms such as palmoplantar pustulosis (PPP) and peripheral and/or axial articular manifestations [1]. For the treatment of SAPHO syndrome, nonsteroidal anti-inflammatory drugs (NSAIDs), glucocorticosteroids, antibiotics, bisphosphonates, and DMARDs have been reported to be effective to varying degrees. In the last decade, a number of reports documented the successful use of biological treatments for this rare disease, mainly with TNF-*α* inhibitors, which have substantial efficacy for both cutaneous and articular manifestations. However, the application of this type of drug is contraindicated in cases associated with demyelinating nervous diseases such as MS, Guillain–Barre syndrome and chronic inflammatory demyelinating polyneuropathy (CIDP) because of the potential risk of aggravating those conditions. Here, we describe a case of SAPHO syndrome complicated by lesions of the central nervous system resembling MS which was successfully treated with brodalumab, an IL-17 receptor blocker. Peripheral and axial manifestations and the serum inflammatory response improved promptly after the initiation of therapy. Efficacy was maintained until the 50th week of observation with no deterioration of the central nervous system lesions and no major complications from the therapy.

## 2. Case Presentation

**Figure 1:**
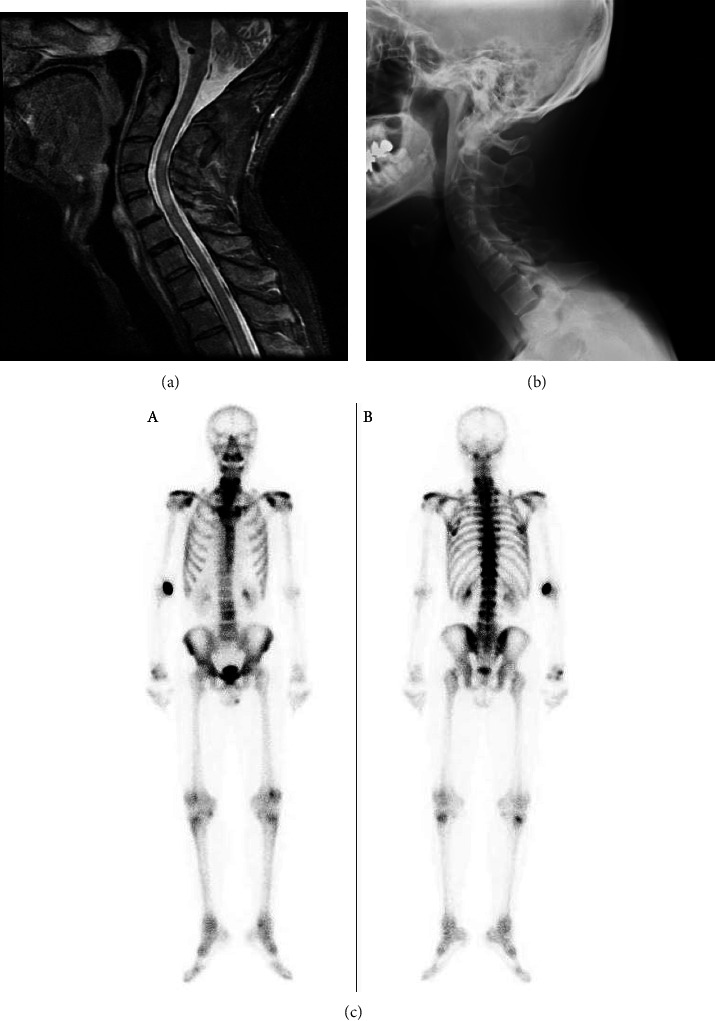
Radiographic images at first visit: (a) T2WI MRI image of the neck, (b) cervical X-ray, and (c) ^99m^Tc-HMDP scintigraphy, ventral view (A) and dorsal view (B). Significant accumulation in the wrists, knees and sternoclavicular joints is shown.

**Figure 2:**
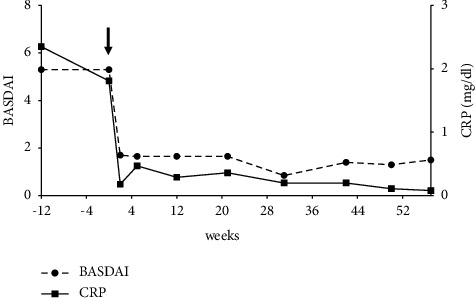
Changes over time in BASDAI and CRP during the therapy with Brodalumab. At 0 weeks, subcutaneous injection of Brodalumab 210 mg was initiated (arrow) and repeated weekly for two weeks. After loading, it was maintained by injection every two weeks.

A 39-year-old Japanese man was referred to our department with pains in his neck and anterior chest. He had started to suffer from pain in his neck thirteen years ago and gradually felt difficulty with cervical rotation. He had been diagnosed with palmoplantar pustulosis (PPP) eight years before. He had also been suffering from optic neuritis for seven years. Before his visit to us, he was referred to our department of neurology for cervical chord lesions detected incidentally by MRI (Figure 1(a)). Thorough medical examination including a neurological assessment revealed a mild increase in neutrophils in the cerebrospinal fluid (CSF). Although the neurological symptoms and findings failed the criteria for MS, his history of optic neuritis and the CSF abnormality coupled with the T2WI cervical chord lesions shown on the MRI suggested possible MS. He was referred to our department for a musculoskeletal evaluation, which revealed joint swelling in both knees and wrists. Multiple pustules were observed in his feet and palms, which is compatible with PPP. Right and left neck rotations were restricted to 38° and 44°, respectively. Occiput to wall distance was 15 cm (normal: 0 cm) and Schober's test gave 3 cm (normal >5 cm), indicating that axial movement was restricted. On the radiographic image, the third to fifth cervical bones were assimilated (Figure 1(b)) while an MRI of the sacroiliac (SI) joint was almost normal. ^99m^Tc-HMDP bone scintigraphy showed accumulation in the wrists, knees, and sternoclavicular joints (Figure 1(c)). In transthoracic echocardiograph, the cardiac function was preserved without heart valvular disease. No conduction abnormality was detected in the electrocardiogram. Ophthalmological examination excluded complication of eye disorders such as uveitis, scleritis, and conjunctivitis. Collectively, from these articular manifestations and characteristic skin symptoms, he was diagnosed with the SAPHO syndrome. He had been treated with prednisolone, methotrexate (maximum dose: 14 mg/week), and tacrolimus. However, these therapies had not led to remission. After our examination, salazosulfapyridine was added; however, no significant effect was obtained after two months. In the meantime, disease activity stayed consistently high as shown in the Bath-Ankylosing Disease Activity Index (BASDAI) of 4-5 and a CRP of 3–5 mg/dl. As treatment with the DMARDs was unsuccessful, the application of a biologic agent was considered. As a TNF-*α* inhibitor was contraindicated due to the complication of the central nervous system lesions, brodalumab, which is an IL-17 receptor blocker, was administered with the informed consent of the patient. Before initiation of the therapy, he was confirmed to be free from inflammatory bowel disease from his medical history and free from any fungal infection from his serum *β*-D-glucan levels. Clinical symptoms including the severe neck pain were resolved immediately after the introduction of brodalumab. BASDAI and CRP improved from 5.1 to 2.3 mg/dl before treatment to 1.3 and 0.2 mg/dl respectively at four weeks after treatment (Figure 2). The clinical improvement was maintained until the 50th week of treatment with brodalumab. No radiographic progression in the cervical vertebrae or peripheral joints was observed. The effect of brodalumab on the cutaneous symptoms of SAPHO syndrome was not clear in this case, because they had improved spontaneously before the initiation of biologic intervention. No recurrence or deterioration of the central nervous system lesions was observed during the period. Similarly, no adverse events from the IL-17 blockade such as inflammatory bowel disease, mucocutaneous candidiasis, or neutropenia were observed.

## 3. Discussion

SAPHO syndrome is an inflammatory disorder, which affects both the skin and joints. Most cases of SAPHO syndrome follow cutaneous manifestations such as PPP. Articular manifestations include both axial and peripheral joints. Although SAPHO syndrome has a resemblance to spondyloarthritis (SpA), it is not currently classified as a SpA such as ankylosing spondylitis (AS), psoriatic arthritis, reactive arthritis, or enteropathic arthritis associated with inflammatory bowel disease. Rather, recent evidence suggests that SAPHO syndrome is better classified on the spectrum of autoinflammatory diseases [2]. SpA patients retain HLA-B27 to a significantly high ratio, while in SAPHO patients, no specific association with HLA has been identified. In this case, HLA-B27 was positive; however, this patient lacked clinical and radiological evidence of inflammation in SI joints or adjacent lumbar lesions. SpA including AS is characteristic of initial inflammation of the SI joint and progressively ascending lumbar lesions. In contrast, he lacked significant SI and lumbar inflammatory lesions, but suffered from cervical hyperostosis and inflammation of the sternoclavicular joints. Thus, he was diagnosed with SAPHO syndrome rather than SpA including AS, irrespective of his HLA-type.

Pathogenesis of SAPHO syndrome has not been elucidated in detail. An excess of cytokines such as TNF-*α*, IL-8, and IL-17 is speculated to play a pathophysiological role in the spectrum of neutrophilic dermatoses associated with SAPHO syndrome, similarly to psoriasis and other autoimmune diseases [3, 4].

To date, the treatment of SAPHO syndrome is performed in a stepwise fashion. NSAIDs are commonly used as a first line treatment for pain relief, but in most cases they are not sufficient. Bisphosphonates have a consistent but transient efficacy against bone inflammation [5]. Immunosuppressive drugs such as cyclosporine, methotrexate, and sulfasalazine have been widely used, demonstrating varying degrees of effectiveness [6, 7]. Recently, biologics have been employed, usually after the failure of conventional medications. Various reports have described the use of anti-TNF-*α* agents such as infliximab and etanercept as therapeutic options for SAPHO syndrome unresponsive or refractory to conventional therapy [8, 9]. These treatments have shown efficacy for joints, bone, and skin manifestations, achieving remission in most cases [10]. On the other hand, there is concern that new onset or exacerbation of demyelinating nervous disorders could be associated with therapies employing TNF-*α* inhibitors [11]. Our case was complicated by central nervous system lesions resembling MS which had developed before the diagnosis of SAPHO syndrome, making the use of TNF-*α* blocking therapy difficult. Another biologic agent which is reported to be effective for SAPHO syndrome is the anti-IL-1*β* drug. It has been reported that the P2X7–IL-1*β* axis became dysregulated in a SAPHO patient [12], supporting the rationale for the use of drugs that target this cytokine. Anakinra, a recombinant IL-1 receptor antagonist, is of proven efficacy in a small number of SAPHO patients [12, 13], although at present, its clinical application is limited.

Human immunological studies identified an increase in the number of Th17 cells in the peripheral blood of SAPHO patients [14]. Recently, a biologic agent targeting IL-23p19 has been shown to be effective for PPP [15], further indicating the possible involvement of the IL-23/IL-17 axis in the pathogenesis of SAPHO syndrome. These findings have raised expectations for the success of IL-17 inhibitors as a therapy for SAPHO syndrome. Several IL-17 targeting agents have been developed for psoriasis or psoriatic arthritis: secukinumab and ixekizumab, which neutralize IL-17A, bimekizumab, which neutralizes IL-17A and IL-17F and brodalumab, which blocks the IL-17 receptor A, resulting in signal blockade of IL-17A as well as IL-17F, IL-17A/F, IL-17C, and IL-17E. A case report has shown the efficacy of secukinumb in improving the skin symptoms for SAPHO syndrome, but not the rheumatic symptoms [16]. In our case, we adopted brodalumab, expecting an additional effect on his articular symptoms as a result of blocking the IL-17 family. Both his peripheral and axial joint symptoms actually regressed, and inflammatory responses such as CRP and ESR normalized promptly after the initiation of brodalumab.

The significance of blocking IL-17F in addition to IL-17A could be supported by a trial with psoriasis. Bimekizumab, a dual blocking antibody for both IL-17A and IL-17F, has completed its phase 3b clinical trial for psoriasis, in which it achieved superior levels of skin clearance compared with secukinumab [17]. Preclinical experiments with bimekizumab using disease-relevant human cells from PsA patients revealed that blocking both IL-17A and IL-17F suppressed in vitro cytokine responses and neutrophil chemotaxis more effectively than IL-17A or IL-17F alone, indicating that IL-17F could also be involved in human chronic tissue inflammation [18]. The additional effect that brodalumab has over secukinumab is further supported by a recent report in which patients with psoriasis who failed to improve with secukinumab or ixekizumab were successfully treated with brodalumab [19].

The limitations of our findings include the fact that it is not clear whether brodalumab was effective for the symptoms except for the articular symptoms such as cutaneous symptoms of SAPHO syndrome and the lesions of central nervous system because in our case, the patient's PPP and the cervical chord lesions in MRI had improved spontaneously before the initiation of brodalumab. The diagnosis process was made from clinical features without histopathological consideration of the skin lesion in this case. Possible appearance of SpA characteristics in the future such as the cardiac and ophthalmological complications should be carefully monitored at follow-up. Regarding the therapeutic response, HLA-B27 positivity itself independent of SAPHO syndrome may confer the immune status that is subject to IL-17 inhibition, which is applied in SpA. It is worth noting that the therapy with brodalumab we employed produced no major complications and no aggravation or recurrence of the central nervous system lesions. Collectively, SAPHO syndrome with HLA-B27 in those patients who are refractory to conventional therapy or for whom TNF-*α* inhibition is contraindicated could be alternatively treated with IL-17 inhibition. Further research into the long-term efficacy and safety of IL-17 targeted therapy for SAPHO syndrome is required before future application. It is worth considering whether in addition to IL-17A, the rest of the IL-17 family could be involved as targets in an optimal therapy for SAPHO syndrome.
